# From algorithms to negotiations: Why health diplomacy must adapt

**DOI:** 10.1371/journal.pgph.0004488

**Published:** 2025-04-23

**Authors:** Brian Li Han Wong, Garry Aslanyan, Warisa Panichkriangkrai, Ricardo Baptista Leite, Jemilah Mahmood, Anders Nordström

**Affiliations:** 1 Global Health Diplomacy Institutional Network, The Health Diplomacy Initiative, Stockholm, Sweden; 2 Department of International Health, Care and Public Health Research Institute, Maastricht University, Maastricht, Netherlands; 3 Department of Global Public Health, Karolinska Institute, Stockholm, Sweden; 4 Dalla Lana School of Public Health, University of Toronto, Toronto, Canada; 5 UNICEF/UNDP/World Bank/WHO Special Programme for Research and Training in Tropical Diseases (TDR), Geneva, Switzerland; 6 International Health Policy Program (IHPP), Ministry of Public Health, Nonthaburi, Thailand; 7 HealthAI, Geneva, Switzerland; 8 Sunway Centre for Planetary Health, Sunway University, Kuala Lumpur, Malaysia; PLOS: Public Library of Science, UNITED STATES OF AMERICA

## Abstract

Health diplomacy traditionally relies on consensus-building across nations, yet the accelerating integration of artificial intelligence (AI) into health systems poses new governance challenges. Rapidly changing geopolitical conditions—exemplified by shifts in U.S. global health funding and the expansion of AI beyond national boundaries—underscore the urgency of rethinking traditional approaches. This paper, based on insights from the Prince Mahidol Award Conference 2025 side meeting on “Navigating the Future: AI & Global Health Diplomacy,” examines how AI can reshape the practice of health diplomacy, both empowering and unsettling global health objectives. We first explore the promise of AI in enhancing disease surveillance, resource allocation, and progress toward universal health coverage. However, inadequate governance can exacerbate inequalities, particularly if AI remains in the hands of profit-focused enterprises or if digital divides persist. Health diplomacy, therefore, must expand its purview to include technical literacy, data ethics, and robust regulatory frameworks that safeguard equity and transparency in AI design and deployment. To illustrate these dynamics, we emphasise the interplay of social, political, commercial, and digital determinants of health, each feeding into AI-driven innovations. Strong diplomatic engagement is critical to ensuring that AI becomes a tool for mutual benefit rather than a catalyst for further fragmentation. Effective policies must integrate environmental sustainability considerations alongside cross-sector collaboration. We conclude that, although AI cannot replace the vital human element of negotiation and trust-building, it can substantially enhance global health outcomes when governed ethically and inclusively. The future of health diplomacy, shaped by AI, requires agile adaptation and unified strategies to preserve equity and planetary well-being.

**Key Messages:**

- Global health is navigating a rapidly changing geopolitical landscape, in which shifts in funding and policy demand agile approaches to AI governance.
- AI can accelerate progress toward universal health coverage but risks widening inequities if not developed and deployed responsibly.
- Diplomacy must evolve beyond traditional forums, requiring new technical fluency and ethical guardrails to manage the impacts of AI on health.
- Effective AI implementation depends on building trust, promoting transparency, and coordinating across multiple sectors and levels of governance.
- Climate considerations must be integrated into AI strategies to ensure that innovations do not undermine planetary health.

A side meeting at the Prince Mahidol Award Conference (PMAC) 2025 brought together experts to discuss “Navigating the Future: AI & Global Health Diplomacy,” spotlighting the urgent need to integrate artificial intelligence (AI) considerations into health diplomacy. A recent commentary in *BMJ Global Health* has similarly underscored how evolving uncertainties—from pandemics to political upheavals—create “opportunity in uncertainty,” demanding novel, multisectoral approaches to global health diplomacy [1]. Although the global health community has long championed cross-border cooperation, the environment is changing rapidly, poignantly illustrated by the recent US pullout from global cooperation on health and climate change [2], and a shift toward a “new age of globalisation” [3], which places additional strains on funding, infrastructure, and diplomacy. In this context, a sharper focus on how AI can help—or potentially hinder—health systems is even more crucial. The swift rise of AI compels a renewed look at how negotiation, governance, and collaboration can safeguard equity and transparency while maximising AI’s potential in public health.

AI is redefining public health, research, and health systems worldwide. Beyond image recognition or disease modelling, AI now underpins core functions in predictive analytics, surveillance, resource allocation, and real-time decision-making. Its influence already stretches from digital health tools facilitating patient triage to large-scale analytics enabling real-time identification of disease outbreaks. However, this new reality intersects with a global health organisational set-up still anchored in diplomacy shaped by trust, personal negotiation, and consensus-building. Its dual nature—tremendous opportunity and significant potential risk—calls for careful cost-benefit analysis, especially given persistent inequalities, shifting geopolitical alliances, and planetary health concerns [4]. Crucially, the greatest promise of AI may lie in transitioning today’s primarily disease-reactive systems toward ecosystems that proactively promote health, quality of life, and well-being.

## The promise and pitfalls of AI in global health

Many envision AI as a powerful tool for accelerating health outcomes, from improving routine immunisation programs to strengthening early warning systems for pandemics. It can detect outbreaks sooner, optimise resource allocation, and help expand universal health coverage by making services more accessible and responsive [4]. However, market forces alone do not guarantee equitable gains [5]. While commercial interests can stimulate progress, a lack of robust governance frameworks heightens the risk that AI will widen rather than narrow existing gaps—both between countries and within them. Even in high-income settings, privileged populations may reap disproportionate benefits, potentially exacerbating inequities. To uphold the principle of equity, responsible AI must be embedded throughout the technology’s lifecycle, from research and development to deployment and evaluation [5].

## Reimagining global health diplomacy for the digital age

Health diplomacy has historically relied on the World Health Assembly for member states to convene and negotiate agreements on critical global health issues but has increasingly expanded to other political and multilateral fora such as the United Nations General Assembly, G7 and G20, and Climate COPs. The underpinnings of these processes are negotiation, compromise, and reciprocity—elements that hinge on interpersonal dynamics, shared norms, and the forging of trust. However, the entry of AI demands expanded skill sets: from technical fluency and data ethics to nuanced awareness of potential algorithmic misuse.

As highlighted by *The Lancet & Financial Times Commission on Governing Health Futures 2030: Growing Up in a Digital World*, bridging the digital divide and embedding human rights in digital innovation are central to shaping a future where AI can enhance global health outcomes [6]. Global health diplomacy, therefore, needs clear ethical guardrails. Creating international norms for AI in health might mirror earlier treaties—such as the Framework Convention on Tobacco Control or the International Health Regulations—but must account for a broader range of issues, including ethics, data ownership, cybersecurity, and sustainability. Existing norms may fail to capture the rapid evolution of machine learning techniques, leaving a patchwork of policies. A consistent, global understanding of different AI ethics and governance frameworks would help mitigate the risk of regulatory arbitrage, wherein private firms or nations bypass more stringent frameworks by operating in jurisdictions with lax oversight [7]. That said, we also acknowledge that a myriad of normative and ethical frameworks exist worldwide which are deeply embedded in national governance structures, making harmonisation efforts particularly challenging and important [8].

## Navigating the AI-Environment nexus

In the era of planetary health, the environmental impact of AI must not be overlooked. Large-scale computing can consume vast amounts of electricity and water [9], prompting debates on low-carbon energy sources, including nuclear [10]. As climate change intensifies, public health diplomacy increasingly intersects with environmental negotiations under the UN Framework Convention on Climate Change or the Convention on Biological Diversity [10]. AI-based solutions may bolster environmental monitoring and policy coherence but could also spike carbon footprints if poorly managed. Without trust in the technology—underpinned by responsible governance—it risks failing to gain the broad adoption needed to fully realise its potential benefits [11].

### Building transparency and trust

Trust underpins cooperation in global health, linking state actors, civil society, and private sector partners. Opaque decision-making, data exploitation, and biases undermine this trust. Early stakeholder engagement, transparent regulations, and community involvement are crucial for acceptance and accountability in digital health [12]. Misinformation and deepfakes also loom large, blurring lines between authentic consensus and manipulated narratives. If diplomats or the public suspect manipulative AI-driven messaging, collaboration can fracture.

Robust frameworks—spanning ethical principles, algorithmic audits, and data privacy safeguards—are vital to maintaining confidence. This was highlighted in a recent article analysing the interconnected nature of health determinants in a digital age, where it became clear that data and AI policies are a core element of governing AI technologies. It also highlighted how the shape of these policies can be significantly affected by the underlying normative and ethical principles of a given country or region [8]. Without such structures, AI adoption suffers, along with the potential gains in cost-effectiveness, workforce strengthening, and targeted interventions for underserved populations.

### Toward a new era of multisectoral collaboration

The interplay of AI and diplomacy requires alliances beyond the usual cast of health ministries and intergovernmental agencies. Modern diplomatic frameworks must adapt to digital governance and AI innovation while maintaining core principles of trust, reciprocity, and equity [13]. Tech companies, academic institutions, philanthropic foundations, and grassroots organisations bring unique resources to the table. Partnerships can spur open-access data-sharing platforms, accelerate capacity-building in low-resource settings, and reinforce accountability. Moreover, building robust communities of practice is essential for sustaining collaborative efforts and facilitating peer learning across diverse stakeholder groups [14]. By coordinating AI initiatives across economic, environmental, and technological sectors, we can break silos and broaden the “Health in All Policies” approach.

Careful navigation of intellectual property rights, data sovereignty, and equitable access remains imperative, as these form key building blocks of AI governance frameworks [15]. New funding mechanisms might encourage public-good-oriented AI research tackling neglected diseases or climate-vulnerable populations. Meanwhile, philanthropic actors could support AI literacy programs in underserved communities, ensuring that breakthroughs serve everyone.

### Charting a path forward

**Fig 1:**
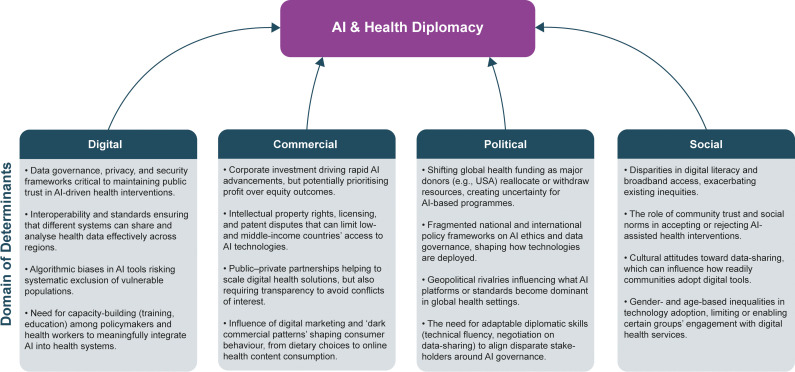
The interplay of digital, commercial, political, and social determinants with AI and health diplomacy.

Several actions are essential for AI to supplement rather than undermine global health diplomacy (Fig 1). First, agile governance structures must adapt to rapid technological shifts. Second, capacity-building should ensure that diplomats and officials appreciate the potential and pitfalls of AI. Third, environmental impact assessments ought to be standard, preventing AI’s resource demands from eroding planetary health. Fourth, broad-based stakeholder engagement can instil trust, transparency, and accountability.

Ultimately, AI is a tool, not a substitute for human interactions. Diplomacy, built on reciprocity and understanding, remains essential to forging agreements that reflect diverse interests and uphold ethical norms [12]. A poorly governed AI landscape could deepen geopolitical rifts and fuel inequalities; a wisely governed one could amplify the core values of diplomacy. The path ahead hinges on whether global health diplomacy can channel the dynamism of AI into equitable, planet-friendly solutions rather than letting technology race beyond our capacity to steward it responsibly.
